# Neural response to repeated auditory stimuli and its association with early language ability in male children with Fragile X syndrome

**DOI:** 10.3389/fnint.2022.987184

**Published:** 2022-11-14

**Authors:** Winko W. An, Charles A. Nelson, Carol L. Wilkinson

**Affiliations:** ^1^Division of Developmental Medicine, Boston Children's Hospital, Boston, MA, United States; ^2^Translational Neuroscience Center, Boston Children's Hospital, Boston, MA, United States; ^3^Harvard Medical School, Boston, MA, United States; ^4^Harvard Graduate School of Education, Cambridge, MA, United States

**Keywords:** Fragile X syndrome, autism, EEG, ERP, phase coherence, neural habituation, language

## Abstract

**Background:**

Fragile X syndrome (FXS) is the most prevalent form of inherited intellectual disability and is commonly associated with autism. Previous studies have linked the structural and functional alterations in FXS with impaired sensory processing and sensory hypersensitivity, which may hinder the early development of cognitive functions such as language comprehension. In this study, we compared the P1 response of the auditory evoked potential and its habituation to repeated auditory stimuli in male children (2–7 years old) with and without FXS, and examined their association with clinical measures in these two groups.

**Methods:**

We collected high-density electroencephalography (EEG) data in an auditory oddball paradigm from 12 male children with FXS and 11 age- and sex-matched typically developing (TD) children. After standardized EEG pre-processing, we conducted a spatial principal component (PC) analysis and identified two major PCs—a frontal PC and a temporal PC. Within each PC, we compared the P1 amplitude and inter-trial phase coherence (ITPC) between the two groups, and performed a series of linear regression analysis to study the association between these EEG measures and several clinical measures, including assessment scores for language abilities, non-verbal skills, and sensory hypersensitivity.

**Results:**

At the temporal PC, both early and late standard stimuli evoked a larger P1 response in FXS compared to TD participants. For temporal ITPC, the TD group showed greater habituation than the FXS group. However, neither group showed significant habituation of the frontal or temporal P1 response. Despite lack of habituation, exploratory analysis of brain-behavior associations observed that within the FXS group, reduced frontal P1 response to late standard stimuli, and increased frontal P1 habituation were both associated with better language scores.

**Conclusion:**

We identified P1 amplitude and ITPC in the temporal region as a contrasting EEG phenotype between the FXS and the TD groups. However, only frontal P1 response and habituation were associated with language measures. Larger longitudinal studies are required to determine whether these EEG measures could be used as biomarkers for language development in patients with FXS.

## 1. Introduction

Fragile X syndrome (FXS) is a genetic condition that affects approximately 1 in 4,000 males and 1 in 8,000 females (Rais et al., 2018). It is the most prevalent form of inherited intellectual disability and is commonly associated with autism (Crawford et al., 2001; Hersh et al., 2011). FXS results from an expansion (>200 repeats for full mutation) and hyper-methylation of a CGG trinucleotide repeat in the *FMR1* (Fragile X messenger ribonucleoprotein 1) gene, and individuals with this mutation exhibit developmental and behavioral challenges including delays in learning, speech and language delay, sensory issues, hyperactivity, and anxiety (NICHD, 2021). The long repeat of the CGG sequence prevents the expression of the encoded FMRP protein, which leads to alterations in the development of synapses, including thin and elongated dendritic spines with increased density, and immature synaptic connections, as evidenced by studies with FXS animal models and postmortem studies of FXS individuals (Rudelli et al., 1985; Hinton et al., 1991; Comery et al., 1997; Irwin et al., 2002). It might also prevent activity-based synapse maturation and synaptic pruning, which are essential in developing normal cognitive functions (reviewed in Schneider et al., 2009; Knoth and Lippé, 2012).

The structural and functional alterations in FXS have been linked with atypical neural processing and arousal modulation problems (Barnea-Goraly et al., 2003). Previous research using rodent models with *FMR1* knockout (KO) mice revealed cortical hyperexcitability due to impaired inhibition and altered neural synchrony (Gonçalves et al., 2013; Zhang et al., 2014). A decreased level of gamma-aminobutyric acid (GABA) receptors and GABAergic input, and increased GABA catabolism were observed in multiple regions in the *FMR1* KO mouse brain (Idrissi et al., 2005; Selby et al., 2007; D'Hulst et al., 2009). This deficit of GABAergic inhibition impacts multiple components of the sensory and cognitive system, including the auditory brainstem (McCullagh et al., 2020), amygdala (Olmos-Serrano et al., 2010), and the auditory cortex (Song et al., 2021), and may underlie the auditory hypersensitivity and auditory processing alterations commonly seen in FXS (Castrén et al., 2003; der Molen et al., 2012a; Schneider et al., 2013; Rotschafer and Razak, 2014). Notably, previous studies in autism have associated auditory processing alterations with language delays (Rincon, 2008; Roberts et al., 2011), a phenotype often shared by the FXS population (Abbeduto et al., 2007; Finestack et al., 2009), which suggests a tight relationship between the auditory response of the brain and language development in individuals with FXS.

Advances in non-invasive neuroimaging techniques like electroencephalography (EEG) have made it possible to track dynamical brain responses, and have become a popular tool for studying auditory brain responses. With EEG collected from the widely-used auditory oddball paradigm, previous studies identified multiple components in the event-related potential (ERP) being altered in individuals with FXS relative to the control group. Elevated N1 (Clair et al., 1987; Castrén et al., 2003; der Molen et al., 2012b; Knoth et al., 2014; Ethridge et al., 2016) and P2 (Clair et al., 1987; der Molen et al., 2012b; Knoth et al., 2014; Ethridge et al., 2016) amplitudes have been consistently reported from individuals with FXS. Habituation of N1, defined as the reduced neural response (i.e., the N1 response) to repeated stimulus presentations, was shown to be weaker in FXS compared to age-matched comparison groups (Castrén et al., 2003; der Molen et al., 2012a; Ethridge et al., 2016). Another canonical ERP component, P1, is relatively less studied in FXS; increased P1 amplitude in FXS was only reported in an animal study (Jonak et al., 2020). In frequency domain, Ethridge et al. (2016) reported increased background gamma (>30 Hz) oscillatory power (baseline-uncorrected), and decreased gamma inter-trial coherence (ITC) and oscillatory power (baseline-corrected) in response to stimuli in individuals with FXS. Nonetheless, most of these studies have focused on the adolescent and adult population; very little is known about the developmental aspect of these neural signatures in FXS.

In typically developing children, Ponton et al. (2000) and Wunderlich et al. (2006) explored the maturation of auditory ERP in infants and young children, and discovered that the waveform and scalp distribution of auditory ERP change as a function of age. The most prominent ERP component in 5–6 years-old children is the P1 with a latency of 80–110 ms (Ponton et al., 2000). Its amplitude decreases by 70–85% by the age of 17 and becomes negligible compared to other components in adults. The maturation of N1 and P2 is the opposite: the magnitude of N1 and P2 components increases with age, and are not reliably measured in children at 5–6 years old, despite being the most dominant features in auditory ERP during adulthood (Ponton et al., 2000). This is possibly the reason why N1 and P2 have been intensively investigated in FXS studies with adult subjects. It also suggests the importance of exploring P1 as a brain signature in children with FXS. This topic had not been visited until recently when Ethridge et al. (2020) examined age-related effects on auditory ERP in a wide age range of individuals with FXS. The authors reported a similar developmental trajectory of P1 amplitude between participants with and without FXS. More specifically, they showed that there was no significant difference in P1 amplitude between groups across age, and that there was a negative correlation between P1 amplitude and age in both groups (Ethridge et al., 2020). This finding raises the question of whether P1 amplitude reduces with repeated stimulus presentations (like N1 amplitude in adults), and whether such neural habituation is a characteristic specific to children with FXS. Additionally, the association between EEG signals and behavioral outcomes in FXS, such as language ability, remains unclear. To our knowledge, only one study by Wilkinson and Nelson (2021) addressed this question, and reported a positive relationship between resting-state gamma power and language scores in male children with FXS.

Understanding the neural mechanisms underlying language and cognitive deficits in FXS is crucial to the development of novel therapies and monitoring effectiveness of therapies in clinical trials. Ideally, therapies would be provided as early as possible; however little research has focused on young children with FXS. This is in part due to difficulties associated with EEG collection in children with behavioral challenges.

To fill these gaps in research, this study recorded EEG data in a passive auditory oddball paradigm from preschool and school aged boys with or without FXS and collected clinical and behavioral measures. First, we compared the amplitude and short-term habituation of the P1 response and its corresponding ITPC between the two participant groups. We hypothesized that the FXS boys would have greater amplitude and less habituation in these measures than their age-matched typically developing peers. Second, to explore the clinical relevance of these EEG measures, we examined how they are associated with language ability, non-verbal skills, and sensory hypersensitivity.

## 2. Materials and methods

### 2.1. Participants

A total of 16 male children (33–78 months old) with Fragile X syndrome (FXS) and 13 age-matched, typically developing (TD) male children (33–80 months old) were recruited for participation. The uneven number of participants between groups was a result of the COVID-19 interruption. All data were collected before the outbreak of COVID-19, and the data collection were paused for almost 2 years. Given uncertainty about the effect of the pandemic on this population, especially as it relates to development, we decided not to wait for additional data collected post-pandemic. In order to reduce possible heterogeneity in neuropathology underlying phenotypic presentation, all FXS participants were male with documented full mutation of the *FMR1* gene. Two individuals had size mosaicism with a combination of full and premutation alleles. Methylation status was not known for all participants; at least 2 had mosaic methylation. As FMR1 is expressed on the X chromosome, females with FXS have variable expression of the *FMR1* gene based on random x-inactivation in different cell types. Given our small sample size, we decided to exclude the single female participant enrolled in the study for this analysis. Additional exclusion criteria across both groups (FXS and TD) included a history of prematurity (< 35 weeks gestational age), low birth weight (< 2,000 g), known birth trauma, known genetic disorders (other than FXS), unstable seizure disorder, current use of anticonvulsant medications, and uncorrected hearing or vision problems. Only children from families whose primary language is English (>50% of the time at home) were included. Some participants were on stable doses of medications [Oxybutin (1 TD); Melatonin (2 FXS); Miralax (1 TD); Sertraline (1 FXS)]. More information about the participants can be found in Table 1.

**Table 1:** Sample characteristics.

	**FXS**	**TD**	
	***N* = 12**	***N* = 11**	***p*-value**
Age, mean in months (SD)	51.9 (16.7)	49.0 (12.8)	0.646
**Maternal education**, ***n*** **(%)**			
< 4-year college degree	0 (0)	0 (0)	
4-year college degree	4 (33.3)	4 (36.4)	
>4-year college degree	8 (66.7)	7 (63.6)	
**Paternal education**, ***n*** **(%)**			
< 4-year college degree	1 (8.3)	0 (0)	
4-year college degree	4 (33.3)	5 (45.5)	
>4-year college degree	7 (58.3)	6 (54.5)	
**Household income**, ***n*** **(%)**			
< $40,000	0 (0)	0 (0)	
$40,000–70,000	2 (16.7)	0 (0)	
$70,000–100,000	3 (25.0)	3 (27.3)	
$100,000–140,000	2 (16.7)	4 (36.4)	
>$140,000	4 (33.3)	4 (36.4)	
Did not answer	1 (8.3)	0 (0)	
**Race**, ***n*** **(%)**			
White	9 (75.0)	6 (54.6)	
African American	0 (0)	1 (9.1)	
Asian	1 (8.3)	1 (9.1)	
Other	2 (12.7)	3 (27.3)	
**Ethnicity**, ***n*** **(%)**			
Hispanic or Latino	2 (16.7)	0 (0)	
**Clinical measures, mean (SD)**			
Nonverbal developmental quotient^*^	56.6 (14.5)	112.5 (16.6)	< 0.001
PLS auditory comprehension (standard score)	67.4 (13.6)	118.6 (9.9)	< 0.001
PLS expressive communication (standard score)	66.2 (15.0)	122.1 (8.8)	< 0.001
VAS receptive language (v-scale score)	8.8 (3.5)	14.6 (1.5)	< 0.001
VAS expressive language (v-scale score)	6.3 (3.5)	15.6 (1.4)	< 0.001
Sensory profile (raw score of 13 selected questions)^**^	24.0 (6.6)	23.6 (5.1)	0.872
Number of trials, mean (SD)
Per condition after preprocessing	61.0 (20.8)	81.3 (12.0)	0.010

* One TD child was excluded for being over 69 months old.

** Two FXS and one TD participants were too young to complete the Sensory Profile questionnaire, and one TD child's caregiver did not fully complete the questionnaire. Their data were excluded. The average scores of four quadrants (seeking, avoiding, sensitivity, registration) are shown in Supplementary Table 2.

PLS, Preschool Language Scales - 5th Edition.

VAS, Vineland Adaptive Behavior Scales - 3rd Edition.

This study was approved by the Institutional Review Board at Boston Children's Hospital/Harvard Medical School (IRB #P00025493). Written informed consent was obtained from all guardians upon their children's participation in the study.

### 2.2. EEG collection and experiment design

The EEG was recorded in a dimly lit, sound-attenuated, electrically shielded room. Participants either sat in their caregiver's lap or sat independently in a chair, high-chair or stroller depending on their preference, and the caregiver was instructed to avoid social interactions or speaking with their child. EEG data were obtained from 12/15 FXS and 12/13 TD participants. An additional TD participant was excluded after artifact rejection (see Section 2.3). In cases where EEG data were not obtained, the child's behavior did not allow for successful net-placement. To facilitate successful net placement, children were provided with a social story with pictures of the lab and EEG net. Children were given the opportunity to see and touch the net, and they could place a practice net on a stuffed animal's head prior to netting. In addition, an extensive interview was completed with the parent to obtain information about whether videos, snacks, toys, music were good distractors or motivators, and what techniques worked best to calm a child down.

**Figure 1:**
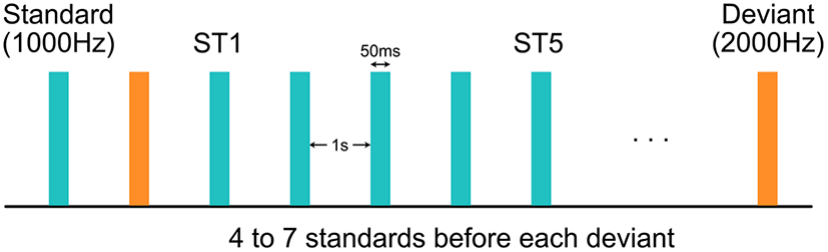
Experimental design of the auditory oddball paradigm. Each standard or deviant stimulus was 50 ms in duration. Inter-stimulus interval was 1 s. The first and the fifth standard stimuli after each deviant stimulus were deemed as the “ST1” and the “ST5” conditions, respectively.

During the experiment, EEG data were collected using a 128-channel HydroCel Geodesic Sensor Net (Version 1, EGI Inc., Eugene, OR) connected to a DC-coupled amplifier (Net Amps 300, EGI Inc., Eugene, OR) with impedance of all electrodes kept below 100 kΩ. Data were sampled at 1,000 Hz with reference to the electrode Cz. A sequence of 800 tones were played with 1,000-ms inter-stimulus interval at 70 dB from a speaker while the child was watching a silent movie for compliance (Figure 1). These tones were all 50 ms in duration, and could be either 1,000 or 2,000-Hz. The 1,000-Hz tones, deemed as the more frequent “standard” (ST) stimuli, appeared for 87.5% of the time, while the 2,000-Hz tones, deemed as the less frequent “deviant” stimuli, accounted for the rest 12.5%. The number of STs preceding a deviant was either 4, 5, 6, or 7, varying with equal probability.

### 2.3. EEG pre-processing

First, videos of participant EEG collection were reviewed and trials where environment- or participant-produced noise interrupted the presentation of stimuli were marked for later exclusion. EEG data were then exported from NetStation (version 4.5, EGI Inc., Eugene, OR) and were batch preprocessed with the Harvard Automated Preprocessing Pipeline for EEG plus Event-Related Software (HAPPE+ER) (Monachino et al., 2022), a MATLAB-based EEG processing pipeline. The processing steps in HAPPE+ER are as follows. Line noise was first removed using CleanLine via a multi-taper regression approach. Signals were then resampled to 250 Hz and low-pass filtered (100 Hz). Bad channels, including those with flat line, residual line noise, and other excessive noise evaluated by a joint probability method, were removed and interpolated. A subsequent wavelet-thresholding artifact removal pipeline (Castellanos and Makarov, 2006)—an algorithm that parses signals into frequency components and identifies artifacts based on the distributions of these components—was implemented to remove noise in the frequency domain. A bandpass filter (1–30 Hz) was then applied to remove slow drifts and high-frequency artifacts. The lower cutoff frequency (i.e., 1 Hz) was chosen to remove excessive slow drifts in several EEG recordings. We suspect these were because of sweating in our subjects due to variable heating in our data collection room and are consistent with low frequency sweat artifacts, especially below 1 Hz, described in Kalevo et al. (2020). When tested, choosing a high-pass filter with a lower cutoff (e.g., 0.1 Hz) included these artifacts into the pipeline, and increased data loss due to trial rejection.

After band-pass filtering, continuous EEG data were then segmented into epochs between 200 ms before and 500 ms after the onset of each stimulus, and were baseline corrected by the average over the pre-stimulus period (−200 to 0 ms). Only those epochs associated with the first (ST1) and the fifth (ST5) standard tone after each deviant were retained in this study. It should be noted that “ST5” was chosen for analysis for two reasons. First, it is more distant from “ST1” than any earlier standards, and thus is expected to elicit more habituation effects. And second, the number of trials in “ST5” (*n* = 92) is closer to the number of trials in “ST1” (*n* = 100) compared to any later standards (*n* < 65). Epochs with residual artifacts, evaluated by their amplitude and joint probability, were removed by HAPPE+ER. We excluded participants with fewer than 20 trials in either ST1 or ST5 condition—12 participants in the FXS group and 11 participants in the TD group remained in this analysis after the overall EEG pre-processing. In order to control for the difference in number of trials between ST1 and ST5, we randomly downsampled the condition with more trials to match with the other condition.

### 2.4. EEG analysis

**Figure 2:**
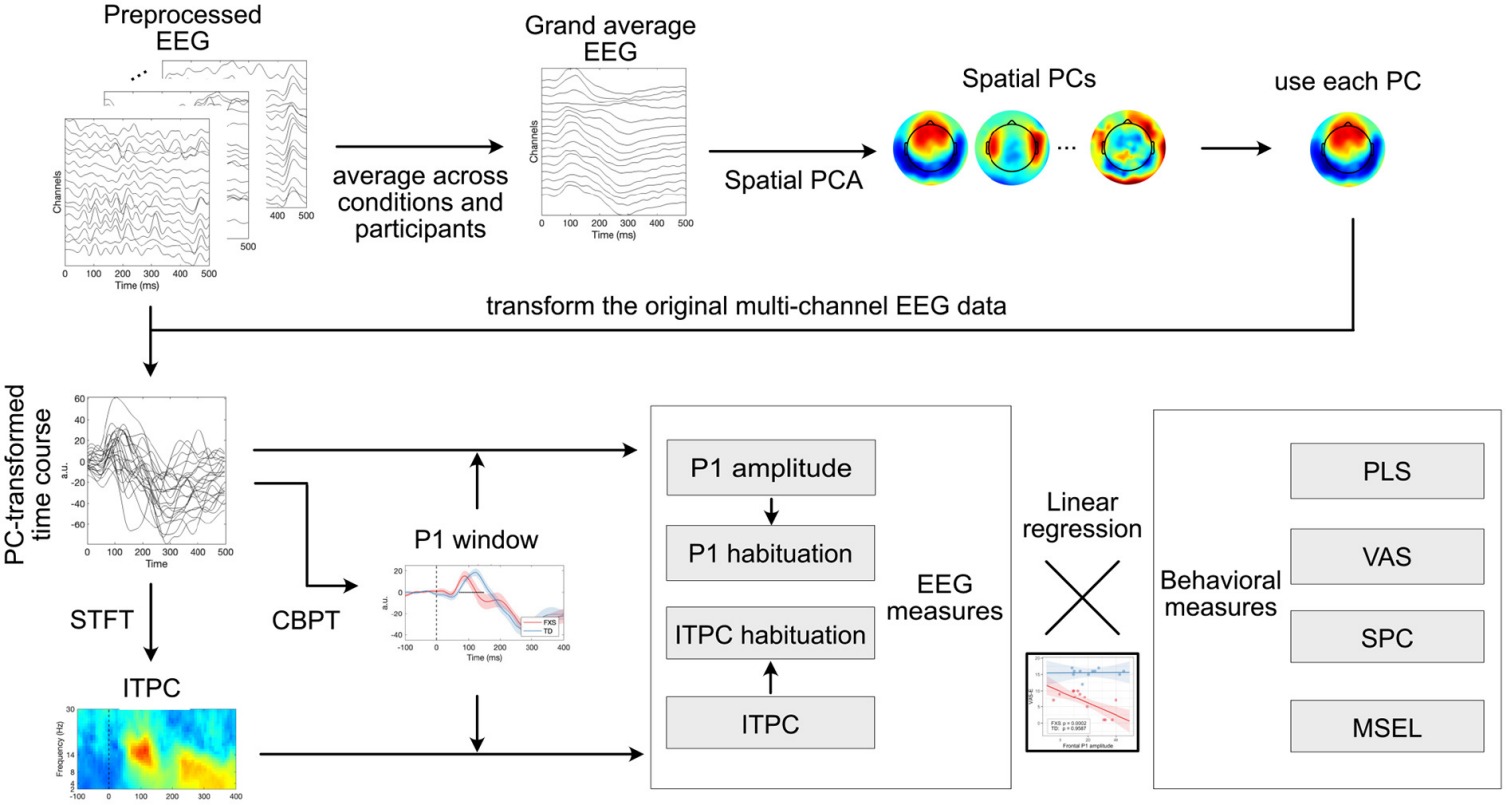
A schematic diagram for all the analyses in this study. For intelligibility, only 16 out of 128 channels are displayed in “Preprocessed EEG” and “Grand averaged EEG”. Each line in “PC-transformed time course” represents the average ERP of an individual. Instantaneous phase was calculated from single trials and then averaged across trials for ITPC. PCA, principal component analysis; a.u., arbitrary unit; STFT, short-time Fourier transform; ITPC, inter-trial phase coherence; CBPT, cluster-based permutation test; PLS, Preschool Language Scales; VAS, Vineland Adaptive Behavior Scales; SPC, Child Sensory Profile; MSEL, Mullen Scales of Early Learning.

An overview of the EEG analysis is shown in Figure 2. In brief, a spatial principal component analysis was performed on preprocessed EEG data, and the principal components were applied on the original multi-channel EEG signals as spatial filters to derive PC-transformed time courses. Event-related potential and inter-trial phase coherence measures were calculated from these time courses, which were later studied for their association with clinical measures of different domains.

#### 2.4.1. Principal component analysis

**Figure 3:**
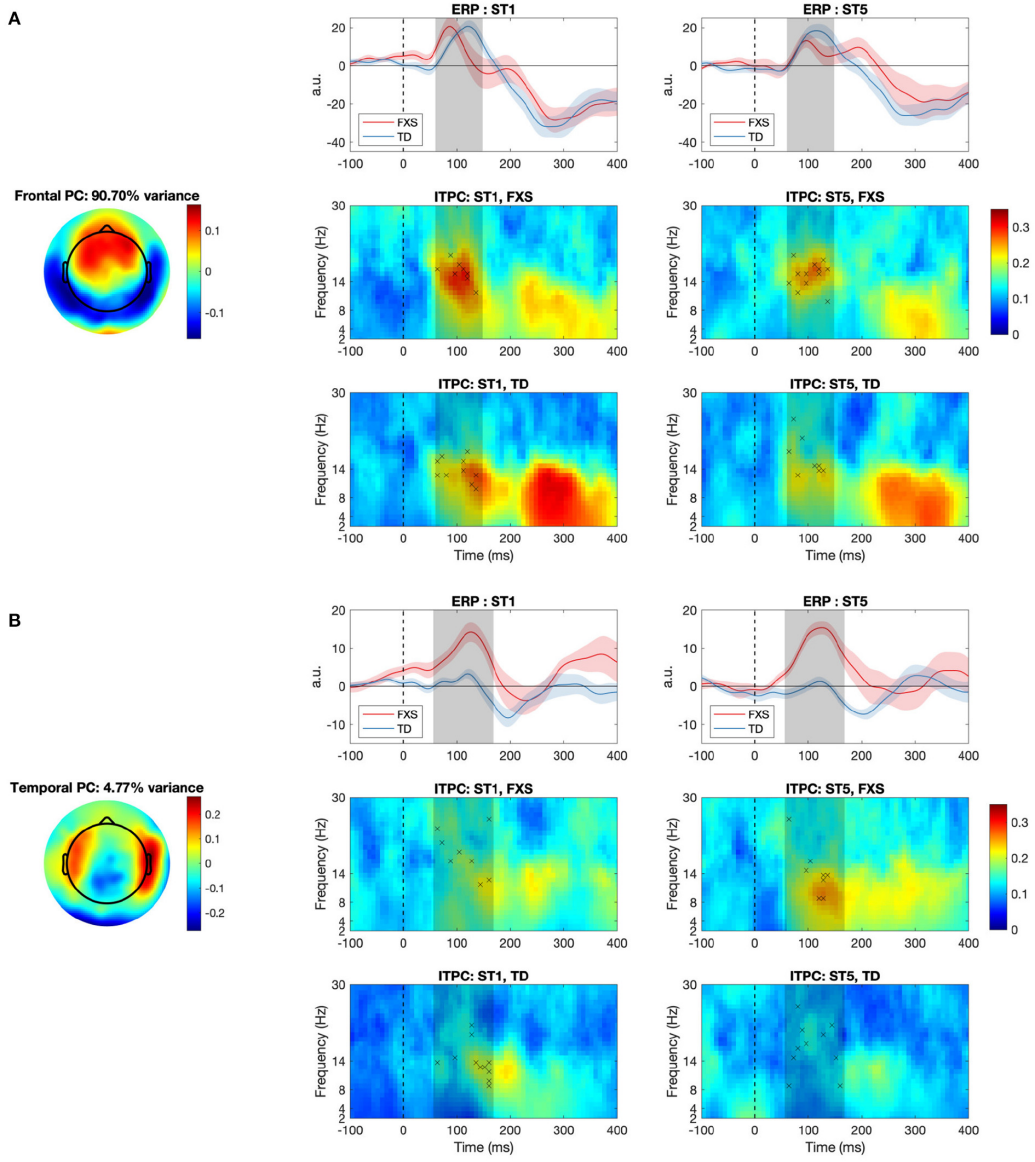
The **(A)** frontal and **(B)** temporal principal component (PC) and their associated event-related potential (ERP) and inter-trial phase coherence (ITPC) for the FXS and the TD group. Dashed lines represent the stimulus onset; the red and blue shaded areas denote standard error of the mean; the gray shaded areas denote the P1 window identified by a non-parametric statistical analysis; crosses in the ITPC plots indicate where maximum ITPC was identified for each participant. a.u., arbitrary unit.

A spatial principal component analysis (PCA) was conducted to identify representative spatial patterns in neural activation following the steps in previous studies (Ethridge et al., 2012, 2015, 2016). EEG data were first averaged across trials, conditions and participants for a grand average EEG matrix, whose dimensions are number of time points by number of channels. A spatial PCA processed each time point as an observation and each channel as a variable, and generated a series of mutually orthogonal principal components (PCs) that could sequentially explain most of the variance in the data. The number of PCs equals the number of time points (i.e., more than 100), and most of them carry a negligible amount of variance explained. In order to determine the number of PCs that should be included for further analysis, we ran a parallel analysis (Franklin et al., 1995) implemented in MATLAB (Shteingart, 2022), which identified two PCs as being significant with more than 95% confidence (Figure 3). We deemed the one with a cluster of high (>0.1) weights in the frontal region as the “frontal PC”, and the one with a cluster of high weights in the temporal regions as the “temporal PC”. All subsequent analyses were performed in these two PCs independently.

#### 2.4.2. Event-related potentials

The PCs derived from the previous step were used as spatial filters to scale and integrate signals from all channels in each trial, for each condition and each participant. This reduced the 128-channel EEG data to one single time course for each trial. Event-related potential (ERP) analysis was conducted with these PC-transformed time courses instead of the original multi-channel signals.

We specifically focused on the P1 component of an ERP in this study, which is the first positive deflection in the average EEG waveform after the stimulus onset. Considering that the ERP waveform changes during childhood (Wunderlich et al., 2006), and that we are uncertain about the exact latency for P1 in children with FXS, we did not use a pre-defined time window for P1 calculation. Instead, we performed a non-parametric cluster-based permutation test (Maris and Oostenveld, 2007) for each condition and group (both cluster-formation and cluster-significance alpha were set at 0.05). This method searched for continuous time intervals in which ERP is statistically above zero at around 100 ms, and we defined the P1 window as the union of such intervals over both conditions and both groups. The P1 window for the frontal PC is 60–140 ms; the P1 window for the temporal PC is 56–168 ms. The amplitude of P1 was then calculated as the peak in each participant's PC-transformed time course within this window. Habituation of P1 was defined as the difference in P1 amplitude between ST1 and ST5 (i.e., ST1–ST5) as we expected to observe a reduced P1 amplitude in ST5 than in ST1.

#### 2.4.3. Inter-trial phase coherence

Inter-trial phase coherence (ITPC) examines the consistency in oscillatory phase across all the trials in a condition. It can be calculated with the following equation:


(1) $$\begin{array}{l} ITPC\left(f,t\right)=|\frac{1}{n}\overset{n}{\underset{i=1}{\sum }}\frac{X_{i}\left(f,t\right)}{\left|X_{i}\left(f,t\right)\right|}| \end{array}$$

where *X*_*i*_(*f, t*) denotes the Short-Time Fourier Transform (STFT) of a given time course *x*(*t*), and *n* denotes the number of trials in a condition. The value of ITPC ranges between 0 and 1, with 0 indicating totally random phase distribution and 1 indicating perfect phase synchronization.

We applied a STFT with a 32-point Hann window, 95% overlap between windows, and a 256-point fast Fourier Transform. We chose a narrow window to capture transient phase changes at the cost of spectral resolution, because the exact frequency of peak phase coherence is not the interest of this study. After a time-frequency map of ITPC was calculated for one condition, we applied the P1 window derived from the ERP analysis in the previous step, and searched for the maximum ITPC value within that time window and across all the frequencies (2–30 Hz)—this is the ITPC measure we used for further analysis. We selected this range of frequencies for calculating ITPC, because, in this study, we are particularly interested in the phase synchrony in the auditory ERPs rather than in the induced responses, and the auditory ERPs are typically represented in low frequency components (below 30 Hz). As in the ERP analysis, habituation of ITPC was defined as the difference in ITPC between ST1 and ST5.

### 2.5. Clinical measures

Receptive and expressive language abilities were evaluated by the Preschool Language Scale - 5th Edition (PLS) (Zimmerman et al., 2011), a comprehensive developmental language assessment standardized for children aged 0–83 months. The Vineland Adaptive Behavior Scales - 3rd Edition (VAS) (Sparrow et al., 2018), a parent-report measure assessing communication, social, motor, and daily living skills commonly used in clinical trials, was also administered. Standard scores and v-scaled scores were used for PLS and VAS, respectively. Non-verbal skills were evaluated by the Mullen Scales of Early Learning (MSEL) (Mullen, 1989), a standardized assessment of development for children 0–69 months of age; a non-verbal developmental quotient (NVDQ) was calculated for all FXS participants and TD participants under 70 months of age based on their fine motor and visual reception scores. One TD child was excluded from NVDQ-related analyses for age. We also included the Child Sensory Profile-2 (SPC) (Dunn, 2014) to evaluate sensory processing patterns. We calculated a customized score for sensory hypersensitivity by summing the raw score of 13 selected questions in the Child Sensory Profile-2 caregiver questionnaire—these questions were picked from the Auditory Processing, Visual Processing and Touch Processing sections, and from the Avoiding and Sensitivity quadrants, which are highly pertinent to the sensory hypersensitivity characteristics. The exact questions selected for SPC calculation are listed in Supplementary Table 1. Two FXS and one TD participants were too young to complete the SPC, and one TD child's caregiver did not fully complete the questionnaire. These subjects were excluded from all SPC-related analyses. A summary of clinical measures is shown in Table 2.

**Table 2:** Clinical measures collected from participants.

**Domain**	**Clinical measures**	**Subtest or scores**
Language	Preschool Language Scales - 5th edition	Auditory comprehension (standard score)
		Expressive communication (standard score)
	Vineland Adaptive Behavior Scales - 3rd edition	Receptive (v-scaled score)
		Expressive (v-scaled score)
Non-verbal	Mullen Scales of Early Learning	Non-verbal developmental quotient
Sensory	Child Sensory Profile-2	13 selected questions (raw score; see Supplementary material)

### 2.6. Linear regression analysis

The association between EEG and clinical measures was explored with a series of linear regression analyses. We used an ordinary least squares (OLS) model to search for a linear relationship between dependent variables (i.e., clinical measures) and independent variables (i.e., EEG measures) interacted with group identity (i.e., FXS or TD). We also included the age of participants as a covariate to parse out any effect of age on EEG or behavior. The equation for this OLS model (Model 1) is as follows:


(2) $$\begin{array}{l} \text{behavior}\sim \text{age}+\text{EEG}+\text{group}+\text{EEG}*\text{group} \end{array}$$

We used the built-in functions in R language (R Core Team, 2020) to calculate the beta coefficient of each term in the model. In cases where the interaction term has a low *p*-value (< 0.25) and the model has a positive adjusted *R*^2^-value, we conducted a marginal effect analysis on the previous OLS model to estimate the brain-behavior association within each group. In cases where the interaction term had a *p*-value of >0.25 and therefore group differences in brain-behavior association was low, the interaction term was discarded to assess whether EEG measures across groups was associated with clinical measures. This simplified OLS model (Model 2) with age and group being covariates is as follows:


(3) $$\begin{array}{l} \text{behavior}\sim \text{age}+\text{group}+\text{EEG} \end{array}$$

With this general framework for regression analysis, we examined the association between each pair of EEG and clinical measures. There are four EEG measures in this study: (1) P1 amplitude of ST5, (2) P1 habituation, (3) ITPC of ST5, and (4) ITPC habituation; and there are six clinical measures: (1) auditory comprehension in PLS (PLS-R), (2) expressive communication in PLS (PLS-E), (3) receptive language in VAS (VAS-R), (4) expressive language in VAS (VAS-E), (5) NVDQ in MSEL, and (6) sensory sensitivity customized score from the SPC. In total, 24 models were estimated for each spatial PC to explore its clinical correlate.

### 2.7. Statistical analysis

We conducted a series of two-tailed two-sample *t*-tests to compare the demographics, clinical measures, and EEG measures within each condition between the FXS and the TD group. Between ST1 and ST5 conditions, we conducted right-tailed paired *t*-tests on EEG measures within each group.

For the linear regression analysis, we applied the False Discovery Rate (FDR) correction to control for family-wise error rate in the estimated marginal effects. FDR corrections were applied for *p*-values in each group (FXS and TD) and domain (language, NVDQ, SPC) separately as they each represent an independent hypothesis. Both unadjusted and adjusted *p*-values are reported.

While we have adjusted for multiple comparisons, we recognize that the sample size for this study is small. We are powered to observe medium- to large-sized effects with the current sample size, and similar effect sizes were observed in resting-state analyses from this same sample (Wilkinson and Nelson, 2021). Findings in this study, especially null findings, should be interpreted under this constraint.

## 3. Results

### 3.1. Sample description

Demographic data, including the MSEL non-verbal developmental quotient (NVDQ), PLS and VAS language scores, and Child Sensory Profile-2 (SPC) scores are shown in Table 1. The FXS and the TD groups are age-matched (*p* = 0.646), but have substantially different NVDQ, and receptive and expressive language abilities (*p* < 0.001 for all). The SPC scores are comparable between the two groups (*p* = 0.872). The TD group has more trials per condition than the FXS group after preprocessing (*p* = 0.010; Table 1). We performed additional correlation analyses to examine the effect of trial numbers on EEG measures. We observed no significant correlation except in temporal ITPC, where it was negatively correlated with trial numbers (*p* = 0.006). However, we did not observe significant correlation within each group for this EEG measure (FXS, *p* = 0.299; TD, *p* = 0.497).

### 3.2. Neural response and habituation

Brain responses to repeated tones within a passive auditory oddball paradigm were analyzed using a spatial principal component (PC) analysis. Two significant PCs were identified in the parallel analysis, one with a cluster of high weights (>0.1) in the frontal region (i.e., the frontal PC; Figure 3A) and the other with a cluster of high weights in the temporal regions (i.e., the temporal PC; Figure 3B). The PC-transformed time courses for the frontal PC show a clear ERP waveform with a P1 peak (60–140 ms) in both FXS and TD. The average ITPC associated with P1 is stronger in ST1 than in ST5 in both groups. In the temporal PC, however, only the FXS group shows a strong P1 response (56–168 ms) in both ST1 and ST5; the TD group does not show a clear P1 peak within the P1 window.

The frontal PC explained more than 90% of the variance in the data, which indicates that the topography of this PC is the dominant pattern observed in almost all the time points. This PC may represent the canonical “auditory evoked response” commonly recorded from medial frontal electrodes, which shares a common shape of waveform among all the subjects and can be observed in all the electrodes with different strengths and/or polarities. The temporal PC, on the other hand, was the most significant pattern when the information about the frontal PC was removed, i.e., in the residual variance of the frontal PC. It explained only less than 5% of the total variance, indicating that this may not be a common effect, but one restricted to a specific condition or a specific group that can be observed strongly only in the temporal electrodes.

**Figure 4:**
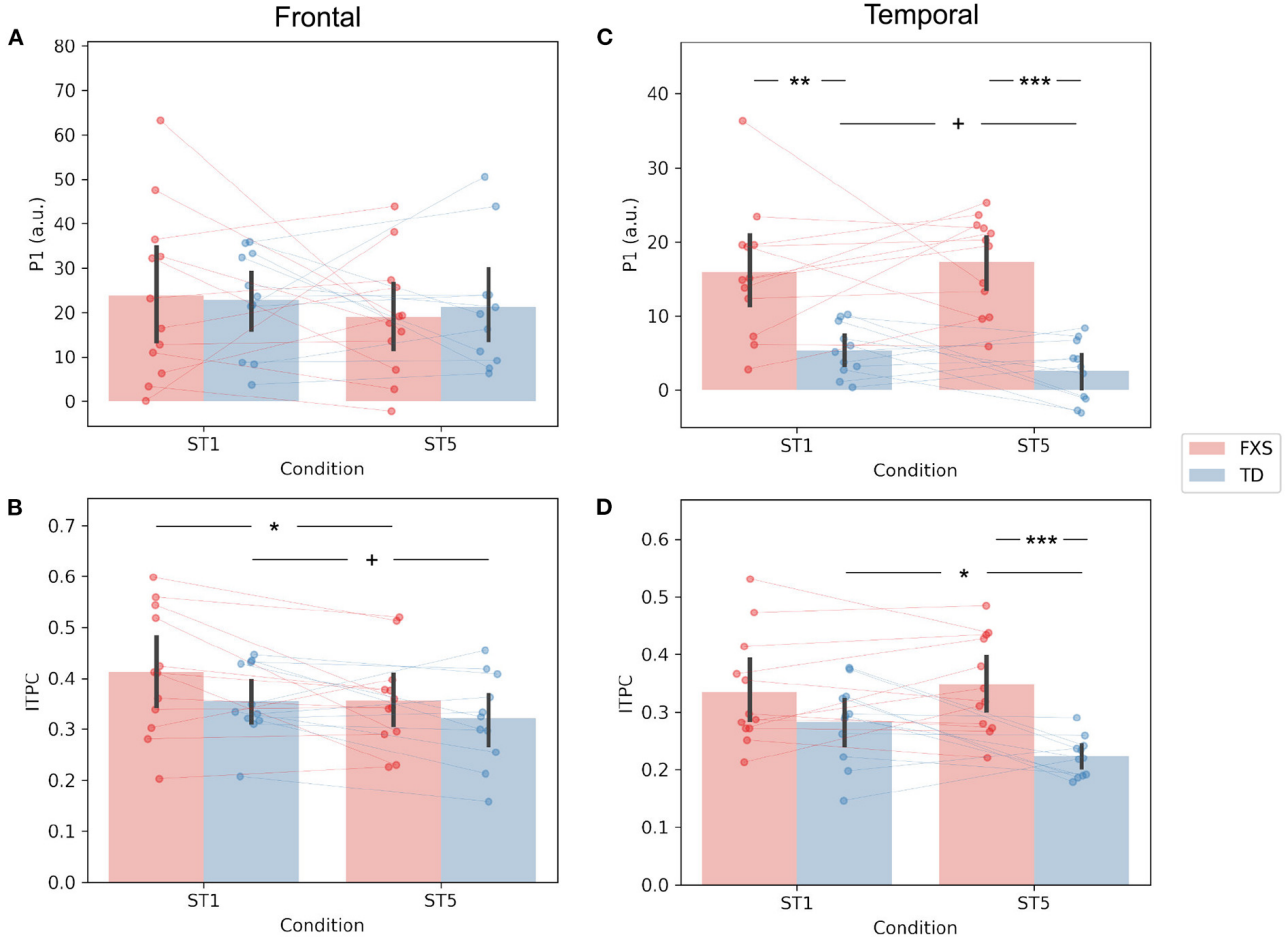
Comparison of **(A)** frontal P1, **(B)** frontal ITPC, **(C)** temporal P1, and **(D)** temporal ITPC between conditions and groups. +*p* < 0.1; *p < 0.05; **p < 0.01; ***p < 0.001.

**Figure 5:**
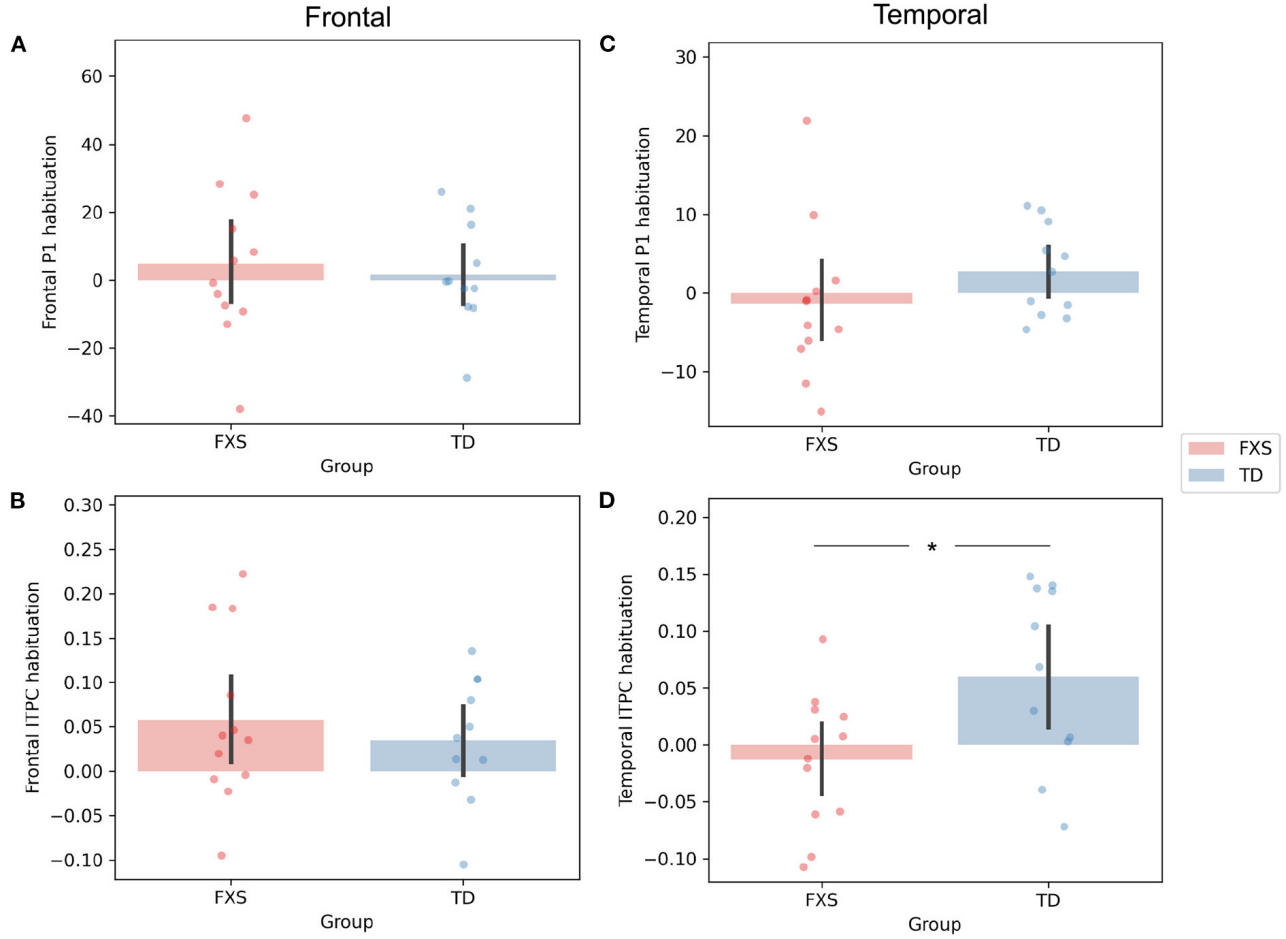
Comparison of habituation (ST1 – ST5) of **(A)** frontal P1, **(B)** frontal ITPC, **(C)** temporal P1, and **(D)** temporal ITPC between conditions and groups. *p < 0.05.

The P1 amplitude in the frontal PC is comparable between FXS and TD in both the ST1 (*p* = 0.889, Cohen's *d* = 0.06) and the ST5 (*p* = 0.705, Cohen's *d* = 0.16) conditions (Figure 4A). No significant difference in P1 amplitude between ST1 and ST5 (i.e., the habituation effect) was identified in either FXS (*p* = 0.239, Cohen's *d* = 0.22) or TD (*p* = 0.370, Cohen's *d* = 0.11) (Figure 4A). The ITPC is also similar between groups in both conditions in the frontal PC (Figure 4B). The habituation effects in ITPC is significant in the FXS group (*p* = 0.031, Cohen's *d* = 0.63) and is trending significant in the TD group (*p* = 0.064, Cohen's *d* = 0.52) group (Figure 4B). Both have individual variability in frontal P1 and ITPC habituation, and there is no significant difference between the groups (Figures 5A,B).

The EEG measures in the temporal PC show a contrasting pattern than those in the frontal PC. The FXS group exhibit a more prominent P1 amplitude than the TD group in both the ST1 (*p* = 0.001, Cohen's *d* = 1.53) and the ST5 (*p* < 0.001, Cohen's *d* = 2.72) conditions (Figure 4C). Their ITPC measures are also significantly different in ST5 (*p* < 0.001, Cohen's *d* = 1.92) (Figure 4D). Given the observed negative correlation between trial number and temporal ITPC, and differences between groups in trial number, a follow-up analysis of covariance showed that the covariate, trial number (*p* = 0.164), did not significantly contribute to the difference in temporal ITPC between groups (*p* = 0.004). Only the TD group showed trending temporal P1 habituation (*p* = 0.072, Cohen's *d* = 0.50) and significant ITPC habituation (*p* = 0.015, Cohen's *d* = 0.80); the TD group had a greater habituation effect in ITPC than the FXS group (*p* = 0.019, Cohen's *d* = 1.06) (Figure 5D).

### 3.3. Relationship between EEG and behavior

We next explored the relationship between EEG and clinical measures through a series of linear regression analyses. Table 3 shows the adjusted *R*^2^ and the *p*-values for EEG × Group interaction in Model 1 (see Supplementary Table 3 for full results). Further analyses of interactions with a *p* < 0.25 and a positive *R*^2^ value are described below. For regressions where the interaction term was >0.25, a simplified regression (Model 2) was performed; no significant associations between EEG and clinical measures were observed (Supplementary Table 4). However, given our small sample size, we are powered to observe statistically significant moderate to large effects, and therefore null findings should be considered with caution.

**Table 3:** Adjusted R^2^ and *p*-values for EEG × Group interaction in Model 1.

	**Adjusted R**^**2**^**/*****p*****-value for EEG** × **Group interaction**					
	**PLS**		**VAS**		**NVDQ**	**SPC**
	**Receptive**	**Expressive**	**Receptive**	**Expressive**		
Frontal P1 habituation (ST1 - ST5)	**0.84 / 0.156**	**0.90 / 0.056+**	**0.65 / 0.136**	**0.83 / 0.063+**	0.76 / 0.408	-0.19 / 0.804
Frontal P1 amplitude (ST5)	**0.86 / 0.056+**	0.91 / 0.008^**^	0.59 / 0.403	**0.80 / 0.077+**	0.76 / 0.261	-0.18 / 0.891
Frontal ITPC habituation (ST1 - ST5)	0.81 / 0.674	0.85 / 0.895	0.44 / 0.817	0.72 / 0.979	0.79 / 0.776	-0.24 / 0.948
Frontal ITPC (ST5)	0.82 / 0.517	0.86 / 0.698	0.46 / 0.391	0.73 / 0.475	**0.77 / 0.164**	-0.15 / 0.592
Temporal P1 habituation (ST1 - ST5)	0.81 / 0.661	0.85 / 0.844	0.46 / 0.779	0.72 / 0.716	0.75 / 0.945	-0.18 / 0.794
Temporal P1 amplitude (ST5)	0.81 / 0.749	0.85 / 0.791	0.46 / 0.912	0.72 / 0.872	0.75 / 0.812	0.03 / 0.484
Temporal ITPC habituation (ST1 - ST5)	0.82 / 0.365	0.85 / 0.637	0.47 / 0.568	0.72 / 0.516	**0.78 / 0.136**	0.15 / 0.025^*^
Temporal ITPC (ST5)	0.81 / 0.835	0.86 / 0.682	0.46 / 0.772	0.71 / 0.889	0.76 / 0.407	-0.12 / 0.250

Models with a positive adjusted R^2^ and a *p*-value < 0.25 for interaction are in bold.

+*p* < 0.1;

* *p* < 0.05;

** *p* < 0.01.

PLS, Receptive: Preschool Language Scales - 5th Edition (PLS), Auditory Comprehension.

PLS, Expressive: PLS, Expressive Communication.

VAS, Receptive: Vineland Adaptive Behavior Scales - 3rd Edition (VAS), Receptive Language.

VAS, Expressive: VAS, Expressive Language.

#### 3.3.1. EEG and language

**Figure 6:**
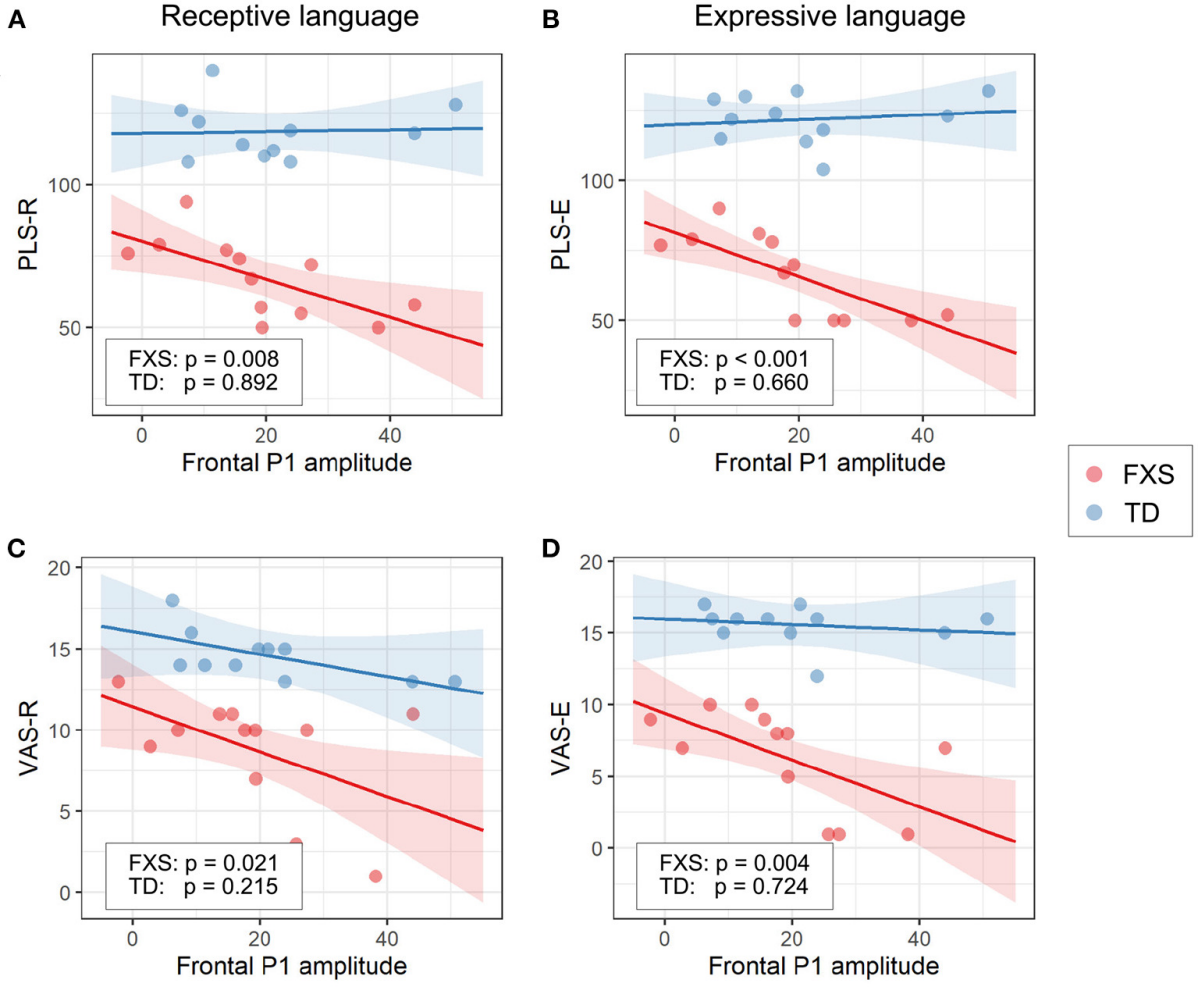
Association between the frontal P1 amplitude of ST5 and **(A)** PLS-R, **(B)** PLS-E, **(C)** VAS-R, and **(D)** VAS-E in Model 1. The lines and shaded areas denote the prediction lines and their 95% confidence interval estimated by the marginal effect. The scattered dots represent individual data. The unadjusted *p*-value of marginal effect for each group is shown in the legend.

**Figure 7:**
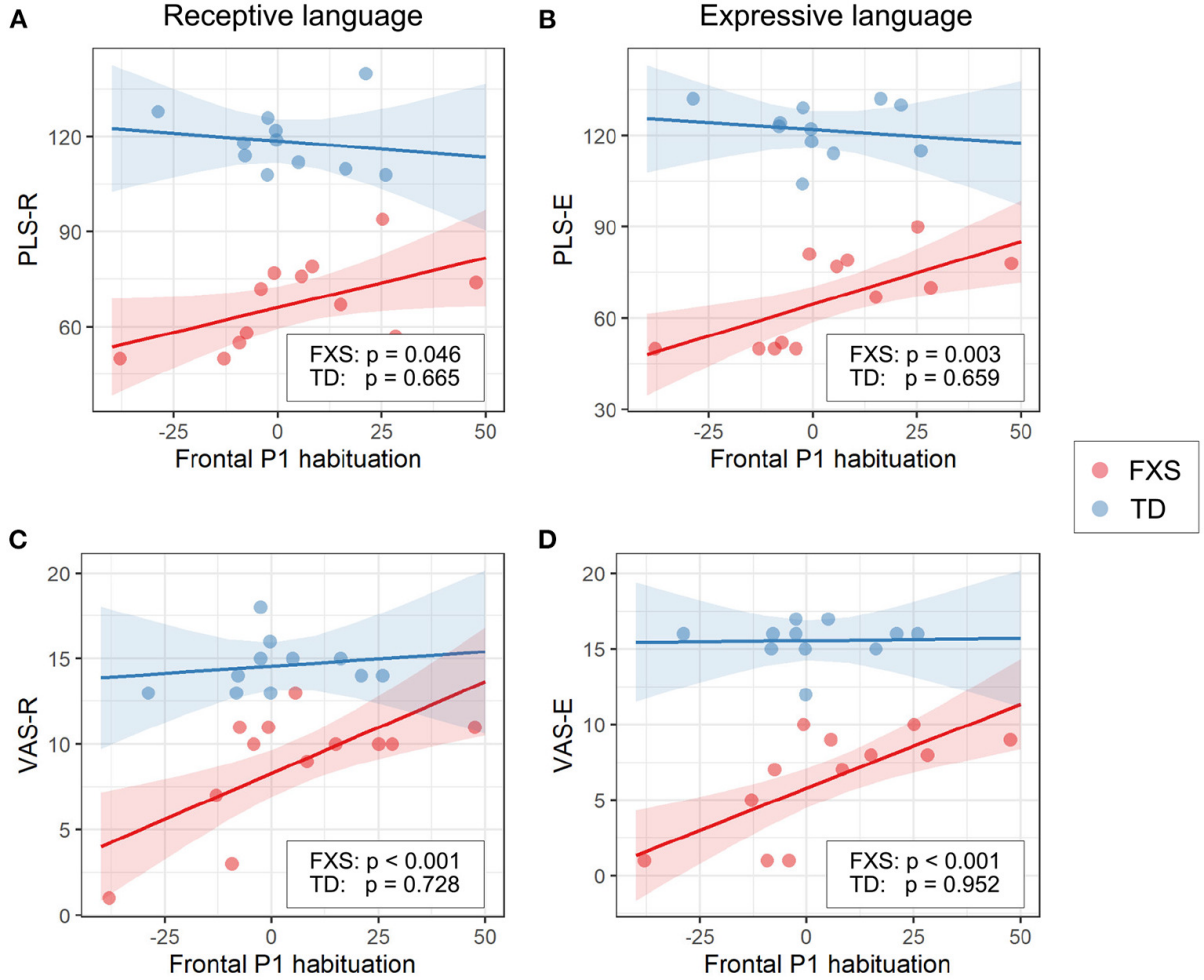
Association between the frontal P1 habituation (ST1 – ST5) and **(A)** PLS-R, **(B)** PLS-E, **(C)** VAS-R, and **(D)** VAS-E in Model 1. The lines and shaded areas denote the prediction lines and their 95% confidence interval estimated by the marginal effect. The scattered dots represent individual data. The unadjusted *p*-value of marginal effect for each group is shown in the legend.

The models for language scores (i.e., PLS-R, PLS-E, VAS-R, and VAS-E) generally show interactions with *p* < 0.25 between frontal P1 measures (i.e., P1 amplitude of ST5 and P1 habituation) and group identity (Table 3), indicating that the two groups may have different P1-language relationships. Accordingly, we analyzed the marginal effects of the model for FXS and TD separately to reveal such group-level differences. Results show that language scores are positively associated with frontal P1 habituation, and negatively associated with frontal P1 amplitude of ST5, only in the FXS group; all *p*-values survived the FDR correction (Table 4). The association is strongest between the P1 amplitude of ST5 and language measures in that a decrease in 1 standard deviation of P1 amplitude (13.5 units) is associated with a 10.5 point increase in the standard score of the PLS-Expressive subscale. In other words, for the average-aged FXS child in our study (4 years, 4 months) with a standard score of 66, a decrease of frontal P1 amplitude by 13.5 units would be associated with an increase in their percentile from the 1st to the 5th, and represent a 6–9 month developmental improvement from a 2.5 to 3 year age-equivalent. These linear relationships are depicted in Figures 6, 7.

**Table 4:** Marginal effects in Model 1 for the association between EEG and language measures in each group.

		**PLS-Receptive**			**PLS-Expressive**			**VAS-Receptive**			**VAS-Expressive**		
		**dy/dx**	***p*-value**	**Adjusted *p*-value**	**dy/dx**	***p*-value**	**Adjusted *p*-value**	**dy/dx**	***p*-value**	**Adjusted *p*-value**	**dy/dx**	***p*-value**	**Adjusted *p*-value**
Frontal P1 habituation (ST1 - ST5)	FXS	0.31	0.046	**0.046**	0.41	0.003	**0.006**	0.11	< 0.001	**0.003**	0.11	< 0.001	**0.002**
	TD	-0.10	0.665	1	-0.09	0.659	1	0.02	0.728	0.970	0.00	0.952	0.952
Frontal P1 amplitude (ST5)	FXS	-0.66	0.008	**0.011**	-0.78	< 0.001	**0.001**	-0.14	0.021	**0.024**	-0.16	0.004	**0.007**
	TD	0.03	0.892	1	0.09	0.660	1	-0.07	0.215	1	-0.02	0.724	1

Significant *p*-values that survived the FDR correction are in bold.

PLS-Receptive: Preschool Language Scales - 5th Edition (PLS), Auditory Comprehension.

PLS-Expressive: PLS, Expressive Communication.

VAS-Receptive: Vineland Adaptive Behavior Scales - 3rd Edition (VAS), Receptive Language.

VAS-Expressive: VAS, Expressive Language.

Since Model 1 shows significant language associations with the P1 amplitude of ST5 as well as with the P1 habituation (ST1–ST5), we completed additional linear regression analyses between language measures and the P1 amplitude of ST1 to determine whether ST1 or ST5 is the dominant factor underlying the association found with the P1 habituation. We did not observe a strong effect in any of these models (results not shown)—the statistics of the group interaction and/or the marginal effect did not meet the thresholds (0.25 and 0.05, respectively) we set for this analysis even before correction for multiple comparisons.

The rest of the EEG measures did not show strong interaction with group, and therefore were analyzed in Model 2. None of the results are significant before correction for multiple comparisons (Supplementary Table 4).

#### 3.3.2. EEG and non-verbal skills/sensory hypersensitivity

**Figure 8:**
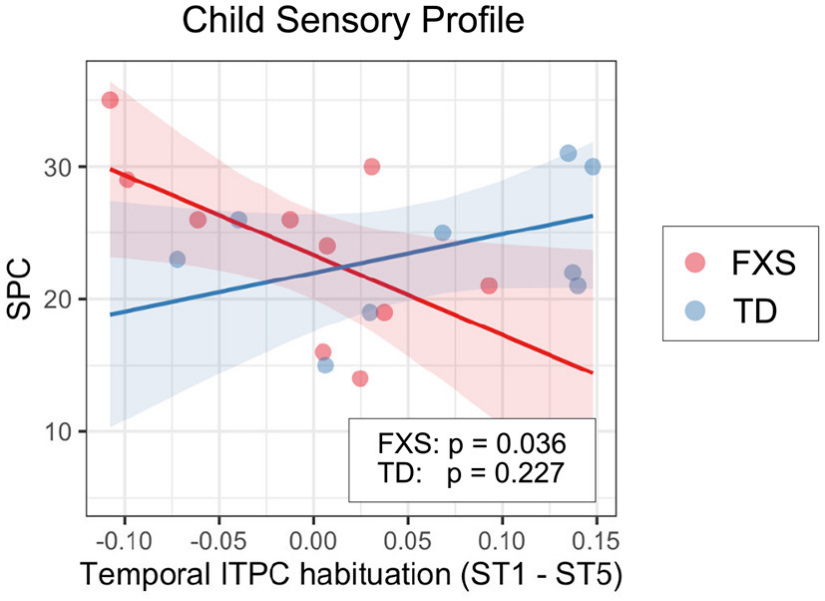
Association between the temporal ITPC habituation (ST1 – ST5) and SPC in Model 1. The lines and shaded areas denote the prediction lines and their 95% confidence interval estimated by the marginal effect. The scattered dots represent individual data. The unadjusted *p*-value of marginal effect for each group is shown in the legend.

We also examined the relationship between EEG and NVDQ or SPC scores. For NVDQ, the linear models for frontal ITPC and temporal ITPC habituation showed possible EEG-Group interactions (*p*-value range 0.025–0.164; Table 3). However, no significant marginal effects were observed in either group or in either EEG measure (Table 5). For SPC, the temporal ITPC habituation was the only EEG measure with a significant interaction with group (*p* = 0.025; Table 3). The marginal effect analysis showed a negative relationship between temporal ITPC habituation and the SPC scores in the FXS (*p* = 0.036), but not in the TD group (*p* = 0.227; Table 5 and Figure 8). None of the results in Model 2 are significant before correction for multiple comparisons (Supplementary Table 4).

**Table 5:** Marginal effects in Model 1 for the association between EEG and non-language measures in each group.

		**NVDQ**			**SPC**		
		**dy/dx**	***p*-value**	**Adjusted *p*-value**	**dy/dx**	***p*-value**	**Adjusted *p*-value**
Frontal ITPC (ST5)	FXS	61.48	0.245	0.245	-	-	-
	TD	-50.81	0.384	0.768	-	-	-
Temporal ITPC habituation (ST1 - ST5)	FXS	-100.88	0.197	0.394	-60.27	0.036	**0.036**
	TD	59.99	0.384	0.384	29.16	0.227	0.227

Significant *p*-values that survived the FDR correction are in bold.

NVDQ, non-verbal developmental quotient; SPC, average raw score of 13 questions selected from Child Sensory Profile - 2.

## 4. Discussion

In this study, we compared the amplitude and habituation of the auditory P1 response and its corresponding inter-trial phase coherence (ITPC) between male children with and without FXS, after performing a spatial principal component analysis (PCA) on their EEG data. We also examined the association between these EEG measures and several clinical measures that assessed sensory sensitivities, language abilities, and non-verbal development. The results show that though the two groups exhibit comparable P1 amplitude and ITPC in the frontal PC, male children with FXS have increased temporal P1 and ITPC. While significant habituation between ST1 and ST5 were not observed for frontal P1 in either group, significant associations between language abilities and frontal P1 habituation were observed in individuals with FXS. However, given our small sample size, brain-language associations should be interpreted with caution.

### 4.1. ERP differences between FXS and TD

The spatial PCA identified two significant PCs in our EEG data—one with high weights in the frontal region, and the other with high weights in the lateralized temporal regions (Figure 3). While we observed significant group differences in the temporal PC which accounted for < 5% of the variance, no group differences were observed in the frontal PC which accounted for the majority of the variance. Interestingly, Ethridge et al. (2020) also found no significant differences in P1 auditory responses in children/adults with FXS compared to typically developmental comparison groups. What is a possible explanation for these spatially dependent findings?

One possibility is that frontal and temporal PCs represent distinct and independent sources of ERP components, and that the observed differences in FXS are specific to one of the sources. Elegant research from Ponton et al. (2002) used dipole source modeling to assess changes in the sources of auditory ERP components across 5–20 years of age. Relevant to our findings, the T-complex, a subcomponent of the auditory ERP commonly recorded in electrodes in the temporal regions, was found to reflect radially-oriented sources hypothesized to originate from the tertiary (parabelt) auditory areas on the lateral surface of the temporal lobe (Näätänen and Picton, 1987; Ponton et al., 2002; Matsuda et al., 2019). Further, T-complex components are independent from the central/frontal auditory ERP generated by tangentially-oriented sources, and have distinct (slower) developmental maturation. Here, we hypothesize that the temporal PC observed in our study is associated with the T-complex, and that the frontal PC is associated with the canonical central/frontal auditory ERP; the increased responses in temporal PC in FXS may represent aberrant responses in the tertiary areas. Given the independence of the T-complex from the central/frontal auditory ERP, similar changes would not necessarily be expected in the frontal PC. Larger studies utilizing source analysis are needed to confirm this hypothesis.

Another possible reason for the spatially different findings could relate to delays in expected developmental shifts in topographic localization of components. Specifically, the topographical location of the N1 peak has been shown to shift from temporal regions prior to the age of 6 (Bruneau et al., 1997) to central regions in older ages (Tonnquist-Uhlen et al., 1995; Knoth and Lippé, 2012). While this has not specifically been studied for P1, given the adjacency of P1 and N1 sources in the auditory cortex (Yvert et al., 2005) and a similar maturation pattern of P1 and N1 (Ponton et al., 2000), it is likely that N1 and P1 have similar change from temporal to central locations with age. Increased temporal P1 in FXS participants could represent delayed maturation (Roberts et al., 2016; Wheeler et al., 2021) resulting in higher P1 in temporal regions.

Finally, our sample size is not well-powered to detect small effects. It is possible that differences between groups are present but larger samples are required to definitely confirm this null finding.

### 4.2. Habituation of ERP in FXS and TD

Contrary to our hypothesis, we did not observe a significant habituation of P1 amplitude in either group (Figures 4A,C). Our hypothesis about P1 habituation originates from previous research where N1 habituation to repeated auditory stimuli was reported in individuals with FXS (Castrén et al., 2003; der Molen et al., 2012a). In addition to the difference in neural signatures between our studies (N1 vs. P1), the participants in these studies were either adults or older children (7–13 years) compared to our cohort. Habituation of auditory response in preschool age range is not well-studied. Given that the waveform of auditory ERP changes throughout childhood (Wunderlich et al., 2006), the results from these studies may not be directly transferable to the current one. More importantly, while N1 and P1 share many commonalities, they are substantially different in their sensitivity to stimulus presentation rate. According to the neural adaptation theory (Kudela et al., 2018), both would reduce their intensity with repeated stimulus presentation. However, P1 would recover to its full amplitude in as fast as a few hundred milliseconds after the stimulus onset (Picton, 2010), while the recovery of N1 takes more than 10 s (Cowan et al., 1993). Since the inter-stimulus interval (ISI) was set at 1,000 ms in this experiment, it likely overlaps with the habituation recovery time of P1, which may lead to increased variability in habituation across individuals (Figures 5A,C). Indeed, we observed substantial frontal P1 habituation in some participants with FXS, and this measure was clinically relevant in our linear regression analysis with language measures. Therefore, the null group-level habituation may not be interpreted as no habituation on the individual level. Future studies with a shorter ISI and/or a bigger sample size are warranted.

### 4.3. Group differences in ITPC and its habituation

In this study, we compared the inter-trial phase coherence (ITPC) of the P1 component and examined their association with clinical measures. We consider this ITPC analysis as complementary to the conventional ERP analysis, because ERP accounts for the “amplitude” aspect of EEG signals, while ITPC considers both “phase” and “frequency”—all three are important characteristics for defining a biomedical signal and are worthy of examination. In the frontal PC, the ITPC measures are comparable between groups, and we observed significant or marginally significant ITPC habituation in both groups. Since cortical phase synchrony is considered to be modulated by cognitive demands (Nash-Kille and Sharma, 2014), these results may have indicated reduced neural activities to repeated sounds in primary and secondary auditory cortex during the P1 time window (see Section 4.1 for more discussions). In the temporal PC, similar to our findings in ERPs, we observed greater ITPC of ST5 in the FXS group than in the TD group; the habituation of temporal ITPC is significant in TD controls, but not in individuals with FXS. These results suggest possible alterations in neural activities in the tertiary cortex in FXS.

It should be noted that the ITPC calculated in this study used a low frequency range (2–30 Hz) and a narrow time window (i.e., the P1 window) in order to specifically examine the phase synchrony of the P1 component. This is different from analyzing the ITPC of oscillatory signals, such as gamma band oscillations, which is also a meaningful topic and was visited in previous studies (Ethridge et al., 2016, 2019), but is out of scope for the current one. It should also be noted that ITPC is relatively less studied than ERP. And, unlike ERP, the habituation characteristics of P1 ITPC have not been well-established in the TD group. The ITPC findings of this study should therefore be interpreted within this context.

### 4.4. Neural correlates with language abilities

In our exploratory analysis of clinical associations, we observed a significant linear relationship between language scores and ST5 frontal P1 amplitude (Figure 6) and frontal P1 habituation (Figure 7) in the FXS group; specifically, weaker P1 response to late standard stimuli (i.e., ST5) as well as larger habituation of P1 was associated with higher receptive and expressive language abilities. Interestingly, no association was observed between ST1 and language abilities (results not shown), suggesting that it is the habituation to repeated tones rather than the general response to tones that impacts language abilities. At the time when ST5 is played, the same standard stimulus has been repeated four times. The level of information novelty in ST5 is extremely low. Therefore, it is cognitively advantageous that our neural system reacts weakly to yet another standard stimulus, so that neural resources can be preserved for other cognitive processes. Huber and O'Reilly (2003) proposed a short-term synaptic depression model to explain this phenomenon. In this model, the response to a recently identified object is suppressed, while any new object, for its high salience, triggers stronger neural activities. This mechanism of neural habituation was considered to aid perceptual processing of a novel object (Huber and O'Reilly, 2003). Later, an EEG study by Jacob and Huber (2020) confirmed the benefit of this habituation mechanism in working memory and novelty detection. Furthermore, a recent behavioral study by Marino and Gervain (2019) linked the novelty detection ability of infants measured at 9 months with their future language outcomes at 12, 14, 18, and 24 months. These previous works suggest a beneficial role of neural habituation in language learning, laying the foundation for understanding our findings in the FXS group. In the TD group, however, we did not observe a strong impact of habituation on language scores. This result could be interpreted from two angles. First, the range of language scores in the TD group is much narrower than that in the FXS group. Thus, it is mathematically more difficult to achieve significance in linear regression models from the TD group, especially given our small sample size (da Silva and Seixas, 2017). Second, the children in the TD group have an intact, unimpaired neural system to support their early language learning. After the critical language period, further language development in these participants may become less sensitive to the cognitive advantages gained from neural habituation, and instead may be driven by other aspects of learning such as language exposure, home support, or non-verbal skills. However, for individuals still in early language acquisition and development (like our study's FXS group), auditory habituation may play a more important role.

The difference in language association between frontal and temporal PCs can possibly be understood via the mapping between PCs and neural sources with different orientations, previously discussed in Section 4.1. The frontal PC in this study may represent the tangentially-oriented sources in the superior surface of the temporal lobe, which incorporates activities mainly from the primary (A1) and the secondary (A2) auditory cortex (Ponton et al., 2002). A1 has a precise tonotopic spatial representation of sound and is critical for processing the physical properties of auditory inputs, including their frequency and intensity. A2 receives projections from A1 and has an important role in the analysis of complex sounds, particularly for human language. Therefore, the functions of A1 and A2 are tightly coupled with multiple aspects of language processing (see Friederici, 2011 for review), and the association between language abilities and frontal P1 measures may reflect the benefit of neural habituation in A1/A2 areas for language learning in individuals with FXS.

The temporal PC in this study has been linked with radially-oriented sources in the temporal lobe, which represents activities mostly in the tertiary cortex (parabelt/A3; Ponton et al., 2002). Unlike A1 and A2, the inner structure and functions of A3 are still relatively unclear (Kaas and Hackett, 2000). Animal and human studies revealed that A3 receives inputs from A2 as well as from other thalamic inputs and is tuned to high-order, abstract stimulus attributes (Kaas et al., 1999; Kaas and Hackett, 2000; Woods et al., 2009). In primates, A3 is interconnected with adjacent portions of the temporal and parietal lobe and with several regions of the frontal lobe (Kaas and Hackett, 2000). Some of these connections are auditory specific, while the others represent a wide range of functions, such as polysensory integration (with visual, motor, somatosensory information), working memory, and stimulus recognition (see Kaas and Hackett, 2000 for review). Therefore, the functional role of A3 on language may be less direct than that of A1 and A2, thus the weak association between language scores and the temporal P1 alterations observed in the FXS group.

In a recent review, Kenny et al. (2022) summarized the EEG studies on FXS in the past decades, and suggested a few EEG features, including the N1 amplitude, gamma oscillatory power, and gamma phase-locking, as promising translational biomarkers for evaluating the efficacy of FXS treatment. In evaluating the potential of EEG biomarkers for use in the prognosis of clinical courses or response to intervention, additional ground work is required (Ewen et al., 2019)—EEG features must be (1) reproducible within an individual across multiple acquisitions, (2) robustly associated with target clinical measures (either concurrent or future depending on biomarker use) and (3) evaluated across a large diverse sample to allow for adjustments based on age, sex, methylation status, etc. Future studies, likely multi-site to increase sample size, are needed to address the above criteria and determine whether P1 amplitude and habituation could be used as a monitoring biomarker for language development. Here, we have demonstrated that passive auditory EEG paradigms can be successfully collected in a young population, and our findings warrant further validation to determine its biomarker potential.

### 4.5. Neural correlates with sensory profile

In this study, we selected 13 questions from the Child Sensory Profile questionnaire and used the sum of their raw scores as the SPC measure for the linear regression analysis. These questions were chosen from the Auditory Processing, Visual Processing and Touch Processing sections, and from the Avoiding and Sensitivity quadrants, which are highly relevant to the sensory phenotypes typically observed in FXS. Surprisingly, no group-level SPC difference was found between FXS and TD, despite sensory hypersensitivity being a common clinical concern in FXS patients. We speculate that this is because Child Sensory Profile is a parental questionnaire, and parents may normalize their scoring based on what they observe in their daily lives, thus lowering scores in the FXS group. However, our analysis also did not use the normed scales of the SPC as we attempted to limit questions to those related to hypersensitivity in auditory, visual, and tactile domains. Direct assessments of sensory hypersensitivity may be more helpful in determining brain differences associated with sensory challenges.

Though there were no group-level differences in the SPC score between groups, within the FXS group we found a significant association between SPC and temporal ITPC habituation; less temporal ITPC habituation is coupled with higher SPC scores or, in other words, more sensory issues. Given that the temporal ITPC habituation is reduced in the FXS group compared to the TD group, we hypothesize that individuals with FXS who have less temporal ITPC habituation with repeated stimuli may have high sensitivity in their tertiary auditory cortex (the source of activities represented by the temporal PC; see Section 4.1 for more discussions), which in turn leads to their high SPC scores. This finding, however, needs to be confirmed in future studies with a larger sample size, as the *p*-value for this association is only moderately below 0.05 (*p* = 0.036).

### 4.6. Limitations

This study is bound to certain limitations. As discussed earlier, the sample size in this study is small and limits our statistical ability to observe smaller but potentially relevant differences in FXS vs. TD children. Further, our lack of observed relationships between EEG and behavioral measures may very well be due to sample size rather than a true finding. Collecting data, especially high-quality EEG data, from children with FXS can be challenging, as they often are sensitive to being touched (especially on their heads), have limited expressive language, and have difficulty sitting in one place. We were successful in obtaining high quality EEG data in 80% of the FXS participants eligible for this study (12/15). To do this, we communicated with parents ahead of visits in order to implement participant-specific behavioral strategies, visual schedules, and positive reinforcements. Careful considerations should always be made to accommodate the needs of these participants, which usually takes a long time for training and accumulating experience. Hence we expect studies with much larger sample sizes to happen through multi-site collaborative research.

Another limitation of this study was the inclusion of only full-mutation males with FXS. Future studies focused on females with FXS are important both clinically and scientifically. While female pathophysiology is likely to be distinct from males due to the differential cell type expression of the FMR1 resulting from X-inactivation, understanding what variables impact phenotypic presentation in the female population can provide important insights to therapeutic intervention. Indeed, in FXS adults, Smith et al. (2021) observed distinct differences in EEG patterns when comparing males and females to sex-matched comparison groups, as well as sex specific brain-behavior associations. Further, identifying biomarkers of language, cognitive, and behavioral outcomes in females is crucial to improving care in this population.

## 5. Conclusion

We analyzed the auditory evoked response to repeated sounds in male children with or without FXS, and examined the neural correlate to early language ability. The P1 amplitude and inter-trial phase coherence in the temporal regions were found to be increased in individuals with FXS compared to their age-matched typically developing peers. Additionally, lower frontal P1 amplitude of late stimuli and higher frontal P1 habituation were shown to be associated with better language abilities in the FXS group. These findings suggest that the auditory P1 might be a potential biomarker for language ability in children with FXS and should be further investigated in larger samples.

## Data availability statement

Data used and/or analyzed to support the findings in this study are available from the corresponding author upon reasonable request.

## Ethics statement

The studies involving human participants were reviewed and approved by Boston Children's Hospital IRB. Written informed consent to participate in this study was provided by the participants' legal guardian/next of kin.

## Author contributions

CN and CW contributed to conception and design of the study. CW recruited participants and collected the data. WA performed the data analysis and data visualization. WA and CW wrote the draft of the manuscript. All authors contributed to manuscript revision, read, and approved the submitted version.

## Funding

Support for this work was provided by FRAXA Research Foundation, Autism Science Foundation, The Pierce Family Fragile X Foundation, Thrasher Research Fund, Society for Developmental Behavioral Pediatrics, Harvard Catalyst Medical Research Investigator Training Award, the Rosamund Stone Zander Translational Neuroscience Center at Boston Children's Hospital, and the National Institutes of Health (1T32MH112510 and 1K23DC017983-01A1).

## Conflict of interest

The authors declare that the research was conducted in the absence of any commercial or financial relationships that could be construed as a potential conflict of interest.

## Publisher's note

All claims expressed in this article are solely those of the authors and do not necessarily represent those of their affiliated organizations, or those of the publisher, the editors and the reviewers. Any product that may be evaluated in this article, or claim that may be made by its manufacturer, is not guaranteed or endorsed by the publisher.
