# Supplementary material for: Neural response to repeated auditory stimuli and its association with early language ability in male children with Fragile X syndrome
Source: Front Integr Neurosci. 2022 Nov 14;16:987184. doi: 10.3389/fnint.2022.987184 (PMC9702328; doi:10.3389/fnint.2022.987184)
Supplement: Supplementary file 1 [file Data_Sheet_1.PDF]

Supplementary Material

Sensory	Quarant	Question
Auditory	Avoiding	Reacts strongly to unexpected or loud noises (for example, sirens, dog barking, hair dryer)
		Holds hands over ears to protect them from sound
		Becomes unproductive with background noise (for example, fan, refrigerator)
	Sensitivity	Struggles to complete tasks when music or TV is on
		Is distracted when there is a lot of noise around
		Tunes me out or seems to ignore me
		Seems not to hear when I call his or her name (even though hearing is OK)
Visual	Sensitivity	Prefers to play or work in low lighting
		Is more bothered by bright lights than other same-aged children
Touch	Avoiding	Shows an emotional or aggressive response to being touched
	Sensitivity	Shows distress during grooming (for example, fights or cries during haircutting, face washing, fingernail cutting)
		Becomes anxious when standing close to others (for example, in a line)
		Rubs or scratches a part of the body that has been touched

Table S1. Questions selected from the Sensory Profile Child questionnaire for calculating the SPC score

	Mean (SD)		p-value
	FXS	TD	
Seeking	47.5 (15.1)	32.4 (8.2)	0.017
Avoiding	38.6 (16.1)	34.6 (7.4)	0.500
Sensitivity	42.8 (13.5)	31.2 (5.5)	0.029
Registration	40.5 (12.8)	33.0 (7.2)	0.140

Table S2. Average scores of four quadrants in Child Sensory Profile

		PLS-R		PLS-E		VAS-R		VAS-E		NVDQ		SPC	
		R2 or beta (SE)	p value	R2 or beta (SE)	p value	R2 or beta (SE)	p value	R2 or beta (SE)	p value	R2 or beta (SE)	p value	R2 or beta (SE)	p value
	Adjusted R2	0.84		0.90		0.65		0.83		0.76		-0.19	
	Variables												
Frontal P1 habituation	Age	-0.11 (0.17)	0.532	-0.24 (0.15)	0.132	0.02 (0.04)	0.500	0.02 (0.03)	0.658	-0.22 (0.28)	0.437	0.02 (0.13)	0.891
(ST1 - ST5)	Group [TD]	52.55 (4.83)***	<0.001	57.34 (4.24)***	<0.001	6.27 (1.00)***	<0.001	9.78 (0.95)***	<0.001	55.63 (7.15)***	<0.001	-1.07 (3.37)	0.755
	EEG	0.31 (0.16)+	0.062	0.41 (0.14)**	0.008	0.11 (0.03)**	0.004	0.11 (0.03)**	0.002	0.19 (0.22)	0.398	-0.09 (0.14)	0.506
	EEG:Group [TD]	-0.41 (0.28)	0.156	-0.50 (0.25)+	0.056	-0.09 (0.06)	0.136	-0.11 (0.05)+	0.063	-0.33 (0.39)	0.408	0.05 (0.18)	0.804
	Adjusted R2	0.86		0.91		0.59		0.80		0.76		-0.18	
	Variables												
Frontal P1 amplitude	Age	-0.06 (0.16)	0.734	-0.19 (0.14)	0.200	0.03 (0.04)	0.519	0.02 (0.04)	0.622	-0.21 (0.28)	0.459	0.06 (0.12)	0.608
(ST5)	Group [TD]	37.79 (8.07)***	<0.001	38.59 (7.03)***	<0.001	4.63 (1.92)*	0.027	6.57 (1.82)**	0.002	42.86 (11.97)**	0.002	-1.35 (6.01)	0.825
	EEG	-0.66 (0.25)*	0.017	-0.78 (0.22)**	0.002	-0.14 (0.06)*	0.033	-0.16 (0.06)*	0.010	-0.31 (0.38)	0.422	0.07 (0.20)	0.730
	EEG:Group [TD]	0.69 (0.34)+	0.056	0.87 (0.30)**	0.008	0.07 (0.08)	0.403	0.14 (0.08)+	0.077	0.58 (0.50)	0.261	0.04 (0.26)	0.891
	Adjusted R2	0.81		0.85		0.44		0.72		0.79		-0.24	
	Variables												
Frontal ITPC habituation	Age	-0.21 (0.18)	0.270	-0.36 (0.18)+	0.060	-0.01 (0.04)	0.884	-0.02 (0.04)	0.653	-0.23 (0.25)	0.371	0.08 (0.11)	0.497
(ST1 - ST5)	Group [TD]	51.64 (6.00)***	<0.001	54.93 (5.83)***	<0.001	5.90 (1.42)***	<0.001	9.05 (1.38)***	<0.001	54.91 (7.39)***	<0.001	0.10 (3.64)	0.978
	EEG	-0.46 (39.33)	0.991	-11.81 (38.25)	0.761	3.02 (9.34)	0.750	-5.51 (9.04)	0.550	65.50 (46.70)	0.179	1.24 (24.12)	0.960
	EEG:Group [TD]	-29.69 (69.34)	0.674	-9.06 (67.45)	0.895	-3.86 (16.47)	0.817	-0.43 (15.95)	0.979	23.76 (82.35)	0.776	-2.47 (37.43)	0.948
	Adjusted R2	0.82		0.86		0.46		0.73		0.77		-0.15	
	Variables												
Frontal ITPC	Age	-0.26 (0.19)	0.191	-0.45 (0.18)*	0.025	-0.01 (0.05)	0.858	-0.03 (0.04)	0.502	-0.36 (0.27)	0.204	0.02 (0.12)	0.849
(ST5)	Group [TD]	64.40 (20.23)**	0.005	63.43 (19.23)**	0.004	9.83 (4.84)+	0.057	12.65 (4.69)*	0.015	91.38 (26.24)**	0.003	6.31 (12.24)	0.614
	EEG	46.16 (41.27)	0.278	51.81 (39.25)	0.203	6.48 (9.88)	0.520	10.55 (9.58)	0.285	61.48 (52.84)	0.261	23.00 (22.76)	0.329
	EEG:Group [TD]	-38.39 (58.05)	0.517	-21.77 (55.20)	0.698	-12.20 (13.89)	0.391	-9.83 (13.47)	0.475	-112.29 (77.20)	0.164	-18.87 (34.44)	0.592
	Adjusted R2	0.81		0.85		0.46		0.72		0.75		-0.18	
	Variables												
Temporal P1 habituation	Age	-0.21 (0.19)	0.281	-0.33 (0.18)+	0.083	0.00 (0.04)	0.976	-0.01 (0.04)	0.855	-0.30 (0.29)	0.311	0.10 (0.11)	0.373
(ST1 - ST5)	Group [TD]	50.56 (5.54)***	<0.001	54.33 (5.42)***	<0.001	5.40 (1.30)***	<0.001	8.84 (1.28)***	<0.001	54.39 (7.42)***	<0.001	-0.58 (3.15)	0.857
	EEG	-0.22 (0.39)	0.583	0.05 (0.38)	0.902	0.04 (0.09)	0.667	0.04 (0.09)	0.656	-0.13 (0.52)	0.800	0.12 (0.23)	0.595
	EEG:Group [TD]	0.35 (0.78)	0.661	0.15 (0.76)	0.844	0.05 (0.18)	0.779	0.07 (0.18)	0.716	0.07 (1.04)	0.945	0.13 (0.49)	0.794
	Adjusted R2	0.81		0.85		0.46		0.72		0.75		0.03	
	Variables												
Temporal P1 amplitude	Age	-0.19 (0.20)	0.349	-0.39 (0.19)+	0.057	-0.02 (0.05)	0.665	-0.03 (0.05)	0.536	-0.37 (0.31)	0.240	0.01 (0.10)	0.901
(ST5)	Group [TD]	52.03 (12.73)***	<0.001	48.94 (12.30)***	<0.001	3.94 (2.96)	0.201	7.86 (2.90)*	0.014	43.15 (18.21)*	0.030	-9.88 (5.90)	0.116
	EEG	0.03 (0.65)	0.967	-0.35 (0.62)	0.587	-0.11 (0.15)	0.468	-0.09 (0.15)	0.532	-0.65 (0.86)	0.461	-0.61 (0.31)+	0.070
	EEG:Group [TD]	-0.38 (1.16)	0.749	0.30 (1.12)	0.791	0.03 (0.27)	0.912	-0.04 (0.26)	0.872	0.42 (1.74)	0.812	0.43 (0.60)	0.484
	Adjusted R2	0.82		0.85		0.47		0.72		0.78		0.15	
	Variables												
Temporal ITPC habituation	Age	-0.18 (0.19)	0.376	-0.33 (0.19)	0.102	-0.01 (0.05)	0.766	-0.01 (0.05)	0.894	-0.27 (0.26)	0.310	0.08 (0.10)	0.445
(ST1 - ST5)	Group [TD]	49.92 (6.00)***	<0.001	54.13 (5.94)***	<0.001	6.01 (1.42)***	<0.001	8.75 (1.40)***	<0.001	51.35 (8.12)***	<0.001	-1.35 (2.81)	0.637
	EEG	-50.32 (62.73)	0.433	-19.77 (62.04)	0.754	-13.59 (14.84)	0.372	-4.19 (14.68)	0.778	-100.88 (78.14)	0.214	-60.27 (28.80)+	0.055
	EEG:Group [TD]	74.62 (80.21)	0.365	38.10 (79.32)	0.637	11.03 (18.97)	0.568	12.45 (18.77)	0.516	160.88 (102.69)	0.136	89.42 (35.69)*	0.025
	Adjusted R2	0.81		0.86		0.46		0.71		0.76		-0.12	
	Variables												
Temporal ITPC	Age	-0.17 (0.19)	0.379	-0.30 (0.18)	0.107	-0.01 (0.04)	0.869	-0.02 (0.04)	0.709	-0.24 (0.27)	0.396	0.10 (0.11)	0.378
(ST5)	Group [TD]	54.72 (31.03)+	0.095	61.93 (29.42)*	0.050	8.67 (7.26)	0.247	8.04 (7.24)	0.281	88.31 (39.76)*	0.040	19.58 (16.41)	0.253
	EEG	-15.44 (44.80)	0.734	-33.59 (42.47)	0.439	8.37 (10.47)	0.435	-1.56 (10.45)	0.883	27.61 (57.38)	0.637	12.46 (23.48)	0.604
	EEG:Group [TD]	-26.66 (126.16)	0.835	-49.88 (119.61)	0.682	-8.69 (29.50)	0.772	4.15 (29.42)	0.889	-137.37 (161.59)	0.407	-81.22 (67.61)	0.250

Table S3. Regression analysis results for Model 1.

+p< 0.1; *p<0.05; **p<0.01; ***p<0.001

PLS-R: Preschool Language Scales - 5th Edition (PLS), Auditory Comprehension; PLS-E: PLS, Expressive Communication; VAS-R: Vineland Adaptive Behavior Scales - 3rd Edition (VAS), Receptive Language; VAS-E: VAS, Expressive Language; NVDQ, non-verbal developmental quotient; SPC, Child Sensory Profile - 2

		PLS-R		PLS-E		VAS-R		VAS-E		NVDQ		SPC	
		R2 or beta (SE)	p-value	R2 or beta (SE)	p-value	R2 or beta (SE)	p-value	R2 or beta (SE)	p-value	R2 or beta (SE)	p-value	R2 or beta (SE)	p-value
Frontal P1 habituation (ST1 - ST5)	Adjusted R2	-		-		-		-		0.76		-0.11	
	Variables												
	Age									-0.24 (0.27)	0.3930	0.03 (0.12)	0.7925
	Group [TD]									54.74 (7.02)***	0.0000	-0.74 (3.00)	0.8094
	EEG									0.09 (0.19)	0.6312	-0.07 (0.09)	0.4768
Frontal P1 amplitude (ST5)	Adjusted R2	-		-		-		-		0.76		-0.11	
	Variables												
	Age									-0.28 (0.28)	0.3223	0.06 (0.10)	0.6069
	Group [TD]									54.15 (7.06)***	0.0000	-0.63 (2.93)	0.8328
	EEG									0.01 (0.26)	0.9755	0.09 (0.12)	0.4418
Frontal ITPC habituation (ST1 - ST5)	Adjusted R2	0.82		0.86		0.47		0.73		0.80		-0.15	
	Variables												
	Age	-0.20 (0.18)	0.2816	-0.36 (0.17)+	0.0532	-0.00 (0.04)	0.9084	-0.02 (0.04)	0.6433	-0.24 (0.24)	0.3349	0.08 (0.11)	0.4788
	Group [TD]	50.42 (5.16)***	0.0000	54.56 (5.00)***	0.0000	5.74 (1.22)***	0.0002	9.03 (1.18)***	0.0000	55.89 (6.39)***	0.0000	-0.02 (3.02)	0.9942
	EEG	-10.01 (31.68)	0.7555	-14.73 (30.68)	0.6366	1.78 (7.50)	0.8152	-5.65 (7.25)	0.4452	73.14 (37.46)+	0.0666	0.29 (18.67)	0.9878
Frontal ITPC (ST5)	Adjusted R2	0.83		0.87		0.47		0.73		-		-0.10	
	Variables												
	Age	-0.26 (0.19)	0.1852	-0.45 (0.18)*	0.0214	-0.01 (0.05)	0.8622	-0.03 (0.04)	0.4993			0.02 (0.12)	0.8733
	Group [TD]	51.47 (5.09)***	0.0000	56.09 (4.80)***	0.0000	5.72 (1.23)***	0.0002	9.34 (1.18)***	0.0000			-0.21 (2.83)	0.9431
	EEG	28.50 (30.99)	0.3693	41.79 (29.24)	0.1692	0.86 (7.48)	0.9094	6.03 (7.21)	0.4135			16.81 (19.29)	0.3972
Temporal P1 habituation (ST1 - ST5)	Adjusted R2	0.82		0.85		0.48		0.73		0.76		-0.11	
	Variables												
	Age	-0.21 (0.18)	0.2652	-0.34 (0.18)+	0.0741	0.00 (0.04)	0.9807	-0.01 (0.04)	0.8445	-0.30 (0.28)	0.2905	0.11 (0.11)	0.3277
	Group [TD]	51.17 (5.26)***	0.0000	54.59 (5.12)***	0.0000	5.49 (1.23)***	0.0003	8.95 (1.21)***	0.0000	54.50 (7.06)***	0.0000	-0.28 (2.85)	0.9227
	EEG	-0.14 (0.34)	0.6926	0.08 (0.33)	0.7996	0.05 (0.08)	0.5110	0.06 (0.08)	0.4724	-0.12 (0.45)	0.7979	0.15 (0.20)	0.4512
Temporal P1 amplitude (ST5)	Adjusted R2	0.82		0.86		0.49		0.74		0.77		0.06	
	Variables												
	Age	-0.20 (0.19)	0.3011	-0.38 (0.18)+	0.0529	-0.02 (0.04)	0.6656	-0.03 (0.04)	0.4986	-0.34 (0.27)	0.2233	0.01 (0.10)	0.8976
	Group [TD]	49.37 (9.50)***	0.0001	51.07 (9.18)***	0.0000	4.15 (2.21)+	0.0754	7.55 (2.16)**	0.0024	46.34 (12.22)**	0.0013	-7.42 (4.73)	0.1376
	EEG	-0.09 (0.53)	0.8730	-0.25 (0.51)	0.6251	-0.10 (0.12)	0.4167	-0.11 (0.12)	0.3869	-0.53 (0.68)	0.4482	-0.51 (0.27)+	0.0812
Temporal ITPC habituation (ST1 - ST5)	Adjusted R2	0.82		0.85		0.48		0.73		-		-	
	Variables												
	Age	-0.20 (0.19)	0.3013	-0.34 (0.18)+	0.0796	-0.02 (0.04)	0.6931	-0.01 (0.04)	0.8116				
	Group [TD]	51.10 (5.85)***	0.0000	54.73 (5.68)***	0.0000	6.18 (1.36)***	0.0002	8.95 (1.35)***	0.0000				
	EEG	-6.41 (41.18)	0.8779	2.64 (40.03)	0.9480	-7.10 (9.60)	0.4684	3.13 (9.52)	0.7460				
Temporal ITPC (ST5)	Adjusted R2	0.82		0.86		0.49		0.72		0.76		-0.15	
	Variables												
	Age	-0.18 (0.18)	0.3263	-0.32 (0.17)+	0.0731	-0.01 (0.04)	0.8039	-0.02 (0.04)	0.7192	-0.29 (0.26)	0.2949	0.07 (0.11)	0.4940
	Group [TD]	48.35 (7.18)***	0.0000	50.02 (6.83)***	0.0000	6.60 (1.68)***	0.0009	9.04 (1.67)***	0.0000	55.51 (9.57)***	0.0000	0.39 (3.84)	0.9209
	EEG	-18.69 (41.01)	0.6538	-39.66 (39.02)	0.3222	7.31 (9.60)	0.4557	-1.06 (9.56)	0.9130	10.88 (53.48)	0.8411	3.79 (22.67)	0.8694

Table S4. Regression analysis results for Model 2.

+p< 0.1; *p<0.05; **p<0.01; ***p<0.001

PLS-R: Preschool Language Scales - 5th Edition (PLS), Auditory Comprehension; PLS-E: PLS, Expressive Communication; VAS-R: Vineland Adaptive Behavior Scales - 3rd Edition (VAS), Receptive Language; VAS-E: VAS, Expressive Language; NVDQ, non-verbal developmental quotient; SPC, Child Sensory Profile - 2
